# Mutations in a TGF-β Ligand, *TGFB3,* Cause Syndromic Aortic Aneurysms and Dissections

**DOI:** 10.1016/j.jacc.2015.01.040

**Published:** 2015-04-07

**Authors:** Aida M. Bertoli-Avella, Elisabeth Gillis, Hiroko Morisaki, Judith M.A. Verhagen, Bianca M. de Graaf, Gerarda van de Beek, Elena Gallo, Boudewijn P.T. Kruithof, Hanka Venselaar, Loretha A. Myers, Steven Laga, Alexander J. Doyle, Gretchen Oswald, Gert W.A. van Cappellen, Itaru Yamanaka, Robert M. van der Helm, Berna Beverloo, Annelies de Klein, Luba Pardo, Martin Lammens, Christina Evers, Koenraad Devriendt, Michiel Dumoulein, Janneke Timmermans, Hennie T. Bruggenwirth, Frans Verheijen, Inez Rodrigus, Gareth Baynam, Marlies Kempers, Johan Saenen, Emeline M. Van Craenenbroeck, Kenji Minatoya, Ritsu Matsukawa, Takuro Tsukube, Noriaki Kubo, Robert Hofstra, Marie Jose Goumans, Jos A. Bekkers, Jolien W. Roos-Hesselink, Ingrid M.B.H. van de Laar, Harry C. Dietz, Lut Van Laer, Takayuki Morisaki, Marja W. Wessels, Bart L. Loeys

**Affiliations:** ∗ Department of Clinical Genetics, Erasmus University Medical Center, Rotterdam, the Netherlands; † Center of Medical Genetics, Faculty of Medicine and Health Sciences, University of Antwerp and Antwerp University Hospital, Antwerp, Belgium; ‡ Department of Cardiology, Erasmus University Medical Center, Rotterdam, the Netherlands; § Departments of Bioscience and Genetics, and Medical Genetics, National Cerebral and Cardiovascular Center, Suita, Osaka, Japan; ‖ McKusick-Nathans Institute of Genetic Medicine, Johns Hopkins University School of Medicine, Baltimore, Maryland; ¶ Department of Molecular Cell Biology, Leiden University Medical Center, Leiden, the Netherlands; # Nijmegen Center for Molecular Life Sciences (NCMLS), Radboud University Nijmegen Medical Center, Nijmegen, the Netherlands; ∗∗ Center for Molecular and Biomolecular Informatics (CMBI), Nijmegen, the Netherlands; †† Department of Cardiac Surgery, Antwerp University Hospital, Antwerp, Belgium; ‡‡ Howard Hughes Medical Institute, Baltimore, Maryland; §§ William Harvey Research Institute, Queen Mary University of London, London, United Kingdom; ‖‖ Erasmus Optical Imaging Centre, Erasmus University Medical Center, Rotterdam, the Netherlands; ¶¶ Department of Pathology, Erasmus University Medical Center, Rotterdam, the Netherlands; ## Department of Bioscience and Genetics, National Cerebral and Cardiovascular Center, Suita, Osaka, Japan; ∗∗∗ Department of Dermatology, Erasmus University Medical Center, Rotterdam, the Netherlands; ††† Department of Pathology, Antwerp University Hospital, University of Antwerp, Antwerp, Belgium; ‡‡‡ Institute of Human Genetics, Heidelberg University, Heidelberg, Germany; §§§ Center for Human Genetics, Leuven, Belgium; ‖‖‖ Department of Cardiology, AZ Groeninge Kortrijk, Kortrijk, Belgium; ¶¶¶ Department of Cardiology, Radboud University Medical Centre, Nijmegen, the Netherlands; ### Genetic Services of Western Australia, Subiaco, Western Australia, Australia; ∗∗∗∗ School of Paediatrics and Child Health, The University of Western Australia, Crawley, Western Australia, Australia; †††† Department of Human Genetics, Radboud University Medical Centre, Nijmegen, the Netherlands; ‡‡‡‡ Department of Cardiology, University Hospital Antwerp, Antwerp, Belgium; §§§§ Department of Cardiovascular Surgery, National Cerebral and Cardiovascular Center, Suita, Osaka, Japan; ‖‖‖‖ Department of Cardiovascular Surgery, Japanese Red Cross Kobe Hospital, Kobe, Japan; ¶¶¶¶ Department of Pediatrics, Urakawa Red Cross Hospital, Urakawa, Hokkaido, Japan; #### Department of Cardio-Thoracic Surgery, Erasmus University Medical Center, Rotterdam, the Netherlands; ∗∗∗∗∗ Department of Pediatrics, Division of Pediatric Cardiology, Johns Hopkins University School of Medicine, Baltimore, Maryland; ††††† Department of Molecular Pathophysiology, Osaka University Graduate School of Pharmaceutical Sciences, Suita, Osaka, Japan

**Keywords:** Loeys-Dietz syndrome, gene, TGF-β pathway, thoracic aortic aneurysm, LAP, latency-associated peptide, LDS, Loeys-Dietz syndrome, LOF, loss of function, MFS, Marfan syndrome, MIM, Mendelian Inheritance in Man, SNP, single nucleotide polymorphism, TAAD, thoracic aortic aneurysms and dissections, TGF, transforming growth factor, TGFBR, transforming growth factor beta receptor

## Abstract

**Background:**

Aneurysms affecting the aorta are a common condition associated with high mortality as a result of aortic dissection or rupture. Investigations of the pathogenic mechanisms involved in syndromic types of thoracic aortic aneurysms, such as Marfan and Loeys-Dietz syndromes, have revealed an important contribution of disturbed transforming growth factor (TGF)-β signaling.

**Objectives:**

This study sought to discover a novel gene causing syndromic aortic aneurysms in order to unravel the underlying pathogenesis.

**Methods:**

We combined genome-wide linkage analysis, exome sequencing, and candidate gene Sanger sequencing in a total of 470 index cases with thoracic aortic aneurysms. Extensive cardiological examination, including physical examination, electrocardiography, and transthoracic echocardiography was performed. In adults, imaging of the entire aorta using computed tomography or magnetic resonance imaging was done.

**Results:**

Here, we report on 43 patients from 11 families with syndromic presentations of aortic aneurysms caused by *TGFB3* mutations. We demonstrate that *TGFB3* mutations are associated with significant cardiovascular involvement, including thoracic/abdominal aortic aneurysm and dissection, and mitral valve disease. Other systemic features overlap clinically with Loeys-Dietz, Shprintzen-Goldberg, and Marfan syndromes, including cleft palate, bifid uvula, skeletal overgrowth, cervical spine instability and clubfoot deformity. In line with previous observations in aortic wall tissues of patients with mutations in effectors of TGF-β signaling (*TGFBR1/2, SMAD3,* and *TGFB2*), we confirm a paradoxical up-regulation of both canonical and noncanonical TGF-β signaling in association with up-regulation of the expression of TGF-β ligands.

**Conclusions:**

Our findings emphasize the broad clinical variability associated with *TGFB3* mutations and highlight the importance of early recognition of the disease because of high cardiovascular risk.

The transforming growth factor (TGF)-β pathway plays an important role in many medically relevant processes, including immunologic maturity, inflammation, cancer, and fibrosis, as well as skeletal, vascular, and hematopoietic homeostasis (1). With the discovery of dysregulated TGF-β signaling in *Fbn1* knockout mice, the TGF-β pathway was revealed as a key player in the pathogenesis of thoracic aortic aneurysm development in Marfan syndrome (MFS; [Mendelian Inheritance in Man (MIM) 154700]) 2, 3. MFS is a multisystemic disease characterized by cardiovascular, ocular, and skeletal features caused by mutations in the *FBN1* gene (4). More recently, we and others identified pathogenic mutations in the genes encoding the TGF-β receptor (TGFBR) subunits TGFBR1 and TGFBR2 5, 6, the signaling transducer SMAD3 (7), the ligand TGFB2 8, 9, and the inhibitor SKI (10), occurring predominantly in patients with syndromic presentations of thoracic aortic aneurysms and dissections (TAAD), designated Loeys-Dietz syndrome (LDS1 [MIM 609192] [11]; LDS2 [MIM 610168] [11]; LDS3 [MIM 613795] [also known as aneurysms-osteoarthritis syndrome] 7, 12, 13, LDS4 [MIM 614816] [8]), and Shprintzen-Goldberg syndrome (SGS [MIM 82212]) 13, 14. The finding of human mutations in a ligand, receptors, a signaling transducer, and an inhibitor of the TGF-β pathway confirms the essential role of TGF-β signaling in aortic aneurysm development.

Recently, de novo mutations in the gene encoding the TGFB3 ligand (*TGFB3*) were reported in 2 girls exhibiting a syndrome affecting body growth (either short or tall stature) accompanied by skeletal features reminiscent of MFS or LDS, but without significant vascular involvement 15, 16, 17. Here, we report that *TGFB3* mutations cause a syndromic form of aortic aneurysms and dissections, characterized by cardiovascular, craniofacial, cutaneous, and skeletal anomalies that significantly overlap with other TGF-β vasculopathies, particularly those within the LDS clinical spectrum.

## Methods

### Patients

All patients or relatives provided written informed consent for participation in this study and, if applicable, publication of photographs. Family 1 was investigated by the department of Clinical Genetics (Erasmus University Medical Center, Rotterdam, the Netherlands) and Center for Medical Genetics (Antwerp University Hospital/University of Antwerp, Belgium) after previous surgical interventions. Clinical geneticists (M.W.W., B.L.L.) examined family members, with special attention to skeletal, joint, skin, and craniofacial features. Medical records from deceased patients were obtained for review. Extensive cardiological examination, including physical examination, electrocardiography, and transthoracic echocardiography, was performed. In adults, imaging of the entire aorta using computed tomography or magnetic resonance imaging was performed. Measurements of the aortic diameter were obtained at the level of the aortic annulus, sinuses of Valsalva, sinotubular junction, proximal ascending aorta, aortic arch, descending aorta, and suprarenal and infrarenal abdominal aorta. An aneurysm was defined as an arterial diameter >1.96 SDs above the predicted diameter 18, 19. Probands from families 2 through 8 and 9 through 11 were referred for molecular and/or clinical evaluation to Antwerp (Belgium) or Osaka (Japan), respectively.

Screening of the entire coding region of *TGFB3* was performed in 470 additional probands (120 probands had whole-exome sequencing), presenting both with syndromic and nonsyndromic forms of TAAD. The majority of these patients had been screened previously for all known TAAD genes. Family members of mutation-positive patients were ascertained and submitted to clinical investigations.

### Genotyping and linkage analysis

**Figure 1:**
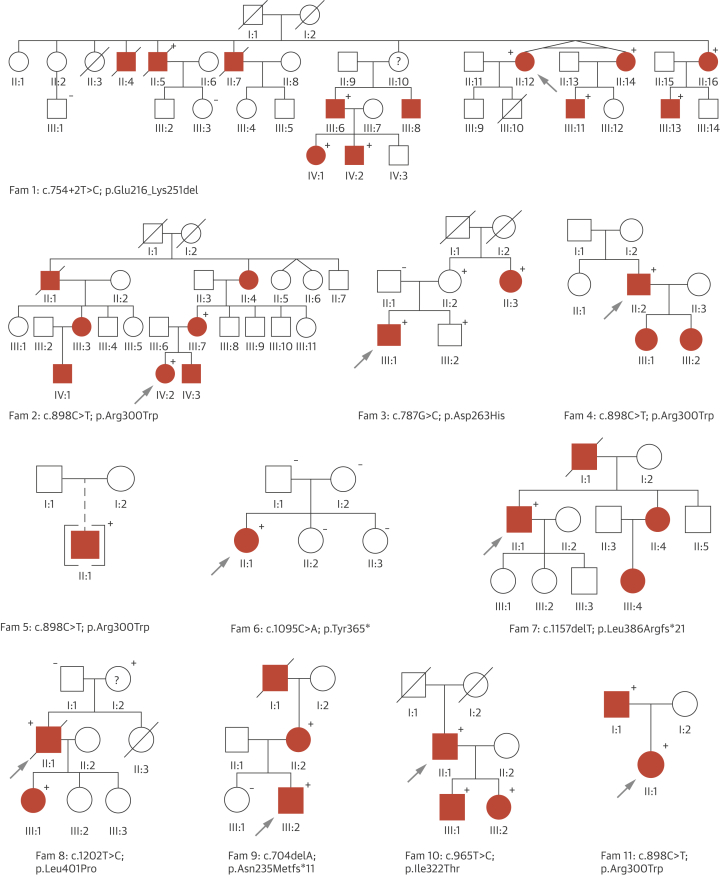
Overview of Families With *TGFB3* Mutations The causal *TGFB3* mutation is shown for each family. Probands are indicated with an **arrow**. **Circle**: female; **square**: male; **open symbol**: unaffected; **solid symbol**: affected; **diagonal line**: deceased; **brackets**: adopted; **question mark**: clinical affection status unknown. **Plus and minus signs** indicate presence or absence of a *TGFB3* mutation, respectively.

Genomic DNA was extracted from peripheral blood samples (Gentra Systems, Qiagen, Hilden, Germany). RNA from 2 patients (1-II:12 and III:11) (Figure 1) was extracted from peripheral blood (collected in PAXgene tubes, PreAnalyliX, Qiagen) according to the manufacturer’s protocol (PreAnalyliX, Qiagen).

Genome-wide genotyping was conducted using DNA from 6 family members (Figure 1, family 1) with Illumina Human SNP-Cyto12 Arrays (Illumina, San Diego, California), containing >262,000 genomic markers, as recommended by the manufacturer. The statistical package, easyLINKAGE Plus v5.08 (20), Merlin v1.0.1 software (Abecasis Lab, University of Michigan), was used to perform single-point and multipoint parametric linkage analysis as previously described 21, 22. Logarithm of odds scores were obtained using a dominant model of inheritance, with 90% penetrance and disease allele frequency of 1:1,000. Allele frequencies of genotyped single nucleotide polymorphisms (SNPs) were set to codominant, and spacing of 0.25 Mb to 0.15 Mb between SNPs was used. Haplotype blocks containing 100 SNPs were constructed with Merlin (option BEST) and they were visualized using HaploPainter (v1.042, H. Thiele, University of Cologne, Germany).

### Sequencing and mutation analysis

Exome sequencing was performed for 120 patients after TruSeq Exome enrichment on HiSeq (Illumina). In 350 other probands, bidirectional Sanger sequencing of exons and exon–intron boundaries was undertaken using polymerase chain reaction primers designed by Primer3 software (v. 4.0.0, S. Rozen, Howard Hughes Medical Institute and the National Institutes of Health, National Human Genome Research Institute) (Online Table 1). Polymerase chain reaction products were purified and sequenced using BigDye Terminator chemistry v3.1 on an ABI Prism3130xl (Applied Biosystems, Foster City, California). Sequences were aligned (SeqScape v2.5 software, Applied Biosystems) and compared with consensus sequences obtained from the human genome databases (Ensembl and NCBI). For annotation of DNA and protein changes, the Mutation Nomenclature guidelines from the Human Genome Variation Society were followed (23). To describe mutations at the cDNA level, the A from the ATG start codon of the reference sequences is numbered as 1 (mRNA NM_003239.2 and protein NP_003230.1).

### In silico analysis of novel variants

The effects of the mutations on protein structure and function were predicted using SIFT BLink (v.5.2.2) and Mutation Taster2. Population frequencies in controls were obtained from dbSNP, Exome Variant Server (EVS) (24), 1000Genomes (25), and Genome of the Netherlands (26). To assess the putative effects on splicing, the Splice Site Prediction by Neural Network (27), the NetGene2 (28) and Alamut Software Suite were used. Protein IDs used for conservation were gi|148342461 (ABQ59024.1), gi|135685 (P17125.1), gi|18266825 (P16047.2), gi|135682 (P17247.1), gi|52138563 (NP_919367.2), gi|410898023 (XP_003962498.1), gi|351050916 (CCD74236.1), gi|17137520 (NP_477340.1), gi|135674 (P01137.2), and gi|48429157 (P61812.1).

### Homology modeling

A homology model was built using the experimentally solved structure of TGFB1 (PDB file [29] 3rjr, 60% identity) as a template. The model was built using an automatic YASARA script (30) with standard parameters. The model contains a homodimer of residues 14 to 412.

### Immunohistochemistry

The protocol for staining of formalin-fixed, paraffin-embedded sections was adapted from Baschong et al. (31) with modifications (Detailed Methods, Online Appendix). Slides were stained overnight at 4°C with anti-pSmad2 antibody (clone A5S, 1:100, Millipore, Billerica, Massachusetts) and anti-pERK1/2 (clone D13.14.4E, 1:100, Cell Signaling Technology, Danvers, Massachusetts) in 0.1% Triton/TBS buffer, washed 3 × 10 min in Perm/Staining buffer, and then stained with anti-rabbit Alexa594 (Molecular Probes, Life Technologies, Carlsbad, California) at 1:200 for 1 h at RT. Slides were then washed 3 × 10 min in Perm/Staining buffer and mounted with Hard Set VECTASHIELD Mounting Media (Vector Laboratories, Burlingame, California) with 2-(4-amidinophenyl)-1H-indole-6-carboxamidine (DAPI). Images were acquired on a Zeiss AxioExaminer (Carl Zeiss, Oberkochen, Germany) with 710NLO-Meta multiphoton confocal microscope at 25× magnification.

### In situ RNA with ACD RNAscope probes

The ACD RNAscope probe Hs-TGFB1 probe (Advanced Cell Diagnostics [ACD], Hayward, California) was used to detect human *TGFB1* transcript in conjunction with the RNAscope 2.0 HD Reagent Kit (RED) from ACD (Detailed Methods, Online Appendix).

### Histology

Slides were histologically examined after hematoxylin-eosin, Elastica van Gieson (elastin), Alcian blue (proteoglycans), or Masson’s trichrome (collagen) staining using standard techniques.

## Results

**Figure 2:**
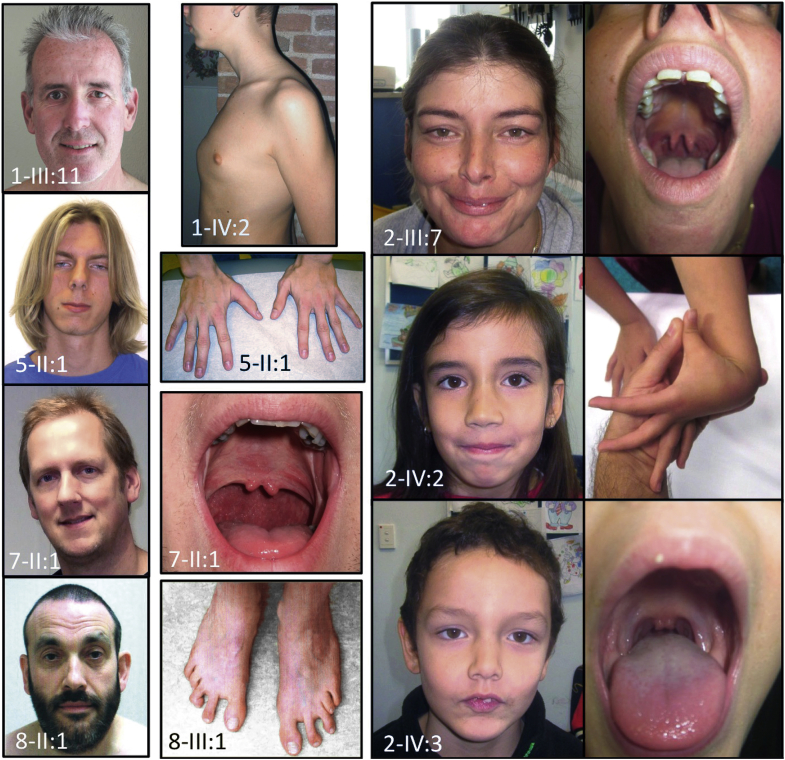
Phenotypic Characteristics of Patients With a *TGFB3* Mutation Observed clinical features include: long face (1-III:11, 5-II:1, 7-II:1, 8-II:1); pectus carinatum (1-IV:2); hypertelorism (2-III:7, 2-IV:2, 2-IV:3, 7-II:1, 8-II:1); bifid uvula (2-III:7, 2-IV:3, 7-II:1); joint hypermobility (2-IV:2); arachnodactyly (5-II:1); and metatarsus adductus (8-III:1). All affected individuals or parents gave permission to publish these photographs.

We studied a large Dutch family (family 1) with clinical features overlapping with MFS and LDS consistent with an autosomal dominant inheritance pattern. Seven family members, between 40 and 68 years of age, presented with aneurysms and dissections, mainly involving the descending thoracic and abdominal aorta (Figure 1, Online Table 2). Three patients died from aortic dissection and rupture of the descending thoracic or abdominal aorta (1-II:4, II:5, and II:7) (Figure 1), confirmed by autopsy in 2 cases (1-II:4, age 57 years and II:5, 56 years). In addition, 4 members had mitral valve abnormalities, ranging from mild prolapse to severe regurgitation requiring surgical intervention. Craniofacial abnormalities were rather subtle, including a long face, high-arched palate, and retrognathia (Figure 2). Pectus deformity and scoliosis were frequently observed (Figure 2). Other recurrent findings included velvety skin, varices, and hiatal hernia. Several family members presented with autoimmune features including (HLA-B27 positive) spondyloarthritis, Graves' disease, and celiac disease.

Sequencing of all known TAAD genes (*ACTA2, COL3A1, EFEMP2, FBN1*, *FLNA, MYH11*, *MYLK, NOTCH1, PRKG1, SKI, SLC2A10, SMAD3*, *TGFB2*, *TGFBR1*, *TGFBR2*) failed to identify a causal mutation. Linkage analysis using SNP genotypes from 6 patients of the family identified 2 large genomic regions on chromosomes 14 and 15 shared by all affected patients (Online Figure 1). Detailed inspection of the genes in the regions identified several candidates, most prominently the *TGFB3* gene on 14q24. Subsequent Sanger sequencing of all 7 exons and intron boundaries identified a heterozygous intronic variant affecting the highly conserved (PhastCons: 1, PhyloP: 4.97) canonical donor splice site of exon 4 (c.754+2T>C), which is absent from variant databases (Variant Server, Genome of the Netherlands, 1000Genomes). Sequencing of the cDNA for 2 patients (1-II:12 and III:11) confirmed skipping of exon 4, leading to an in-frame deletion of 108 nucleotides (Online Figure 2). At the protein level, a deletion of 36 amino acids is expected (p.Glu216_Lys251del). This *TGFB3* mutation (c.754+2T>C) segregated with the clinical phenotype and was also present in 1 young individual (1-IV:1, 17 years old) without documented cardiovascular features (Figure 1, Online Table 2), but with mild systemic manifestations including craniofacial features, easy bruising, and scoliosis.

**Figure 3:**
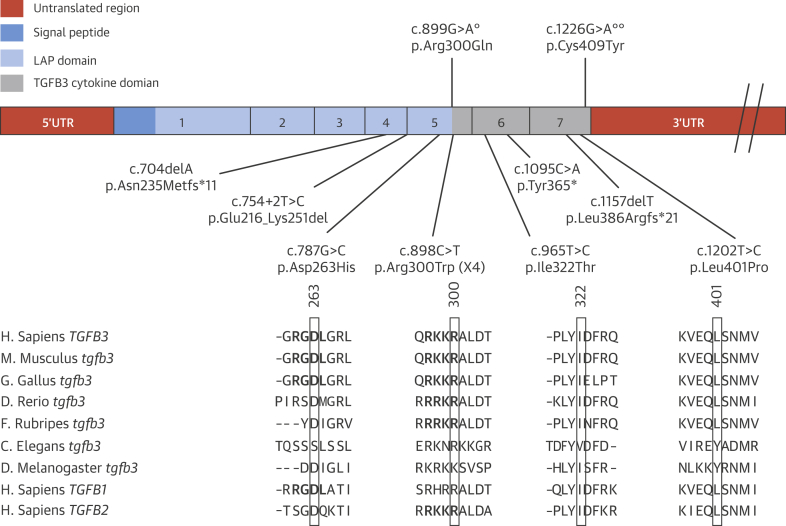
Mutation Overview of the *TGFB3* Gene Exons are represented by **rectangles**. Exon numbering is given, and different colors denote the different protein domains. Mutations found in this study are indicated **below** the gene in the respective domains. Evolutionary conservation in TGFB3 and its related proteins is given for the 4 missense mutations (p.Arg300Trp: Family 2, 4, 5 and 11; p.Asp263His: Family 3; p.Leu401Pro: Family 8; p.Ile322Thr: Family 10). Previously published mutations are shown **above** the gene, with a **single degree symbol** indicating mutation described in Matyas et al. (17) and **double degree symbols** indicating mutation from Rienhoff et al. (16).

To further investigate the role of *TGFB3* in TAAD etiology, DNA samples from 350 syndromic and nonsyndromic TAAD probands were Sanger sequenced for mutations in all exon–intron boundaries and the coding region of *TGFB3.* Additionally, in 120 TAAD patients, a targeted analysis of TAAD candidate genes after whole-exome sequencing was performed. This revealed additional heterozygous *TGFB3* mutations in 10 other probands (7 from Sanger sequencing and 3 from the exome sequencing cohort): 4 different missense mutations, p.Asp263His (family 3), p.Arg300Trp (families 2, 4, 5, 11), p.Ile322Thr (family 10), p.Leu401Pro (family 8); 1 nonsense mutation, p.Tyr365* (family 6); and 2 single-base deletions leading to a frameshift and premature stop codon, p.Leu386Argfs*21 (family 7) and p.Asn235Metfs*11 (family 9) (Figure 3). All missense mutations were predicted as deleterious by SIFT (32) and as disease causing by Mutation Taster (33). The 2 missense mutations in exon 5 (p.Asp263His and p.Arg300Trp) both affect highly conserved amino acids of the latency-associated peptide (LAP) domain, which are also conserved among the TGFB1, TGFB2, and TGFB3 proteins (Figure 3). The p.Asp263His alteration disrupts the Arg-Gly-Asp (RGD) motif, which is essential for binding to the α_v_ß_3_, α_v_ß_6_, α_v_ß_1_, and α_v_ß_5_ integrins 34, 35, 36. Mutations of the RGD motif in LAPβ3 were demonstrated to abolish binding to α_v_ß_3_, α_v_ß_5_, and α_v_ß_6_ (36). The second missense mutation in exon 5, p.Arg300Trp, affects the last amino acid of the LAP domain, disrupting the last residue of the RKKR minimal recognition motif of the furin or related protease cleavage site (37). Mutations affecting similar amino acids in TGFB2 have been shown to be causal in syndromic forms of aortic aneurysms (8). The 2 other missense mutations, p.Ile322Thr and p.Leu401Pro, affect highly conserved amino acids located in the region of the active cytokine. The 3 other mutations create premature stop codons, either in the LAP domain or in the active TGFB3 (cytokine) domain, leading to nonsense-mediated decay or truncated proteins, which probably lose their cytokine activity.

The causal nature of the *TGFB3* mutations was further supported by de novo occurrence (family 6) (Figure 1) and absence from controls (all mutations) in EVS, 1000Genomes, and the Genome of the Netherlands. Although p.Tyr365* in family 6 occurred de novo, we previously identified a *SMAD3* variant (p.Ala250Thr) of unknown significance in the proband. This *SMAD3* variant was also present in the proband’s mother, who presented with variable connective tissue findings and mild cardiovascular involvement, making its precise contribution to pathogenesis unclear.

**Figure 4:**
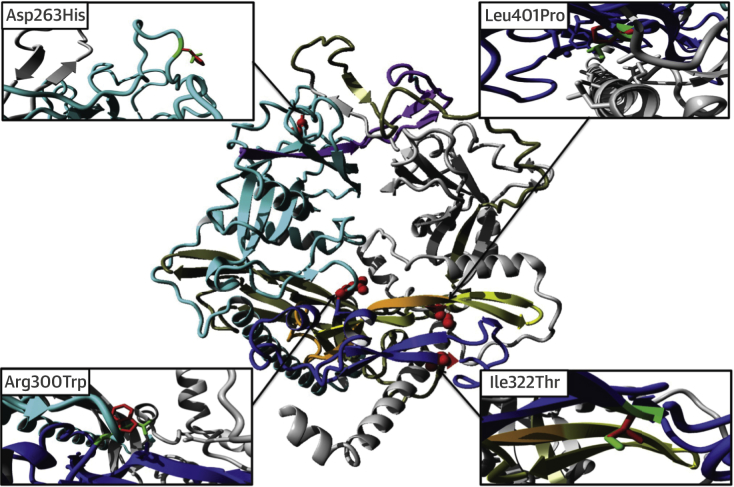
Overview of the TGFB3 Dimer Model and Mutations One monomer is shown in **grey**, with the other monomer in **cyan** (LAP domain) and **blue** (TGFB3 domain). Residues deleted by the p.Glu216_Lys251del mutation are in **purple**. Residues affected by the p.Leu386Argfs21* mutation are in **yellow** (note that this mutation also adds 21 different residues that cannot be modeled). Residues deleted by the p.Tyr365* mutation are shown in **orange** and **yellow** (note that this mutation deletes all residues following Tyr365). Residues deleted by mutation p.Asn235Metfs*11 are shown in **grey-olive** in the second monomer (note that this mutation also adds 11 different new residues, which cannot be modeled). The point mutations p.Asp263His, p.Arg300Trp, p.Ile322Thr, and p.Leu401Pro are shown in **red** with their side chains visible as **red spheres**. A detailed close-up of these mutations is shown in 4 extra panels. In these panels, the wild-type residue side chain is in **green**, whereas the mutant side chain is in **red**. For the p.Arg300Trp mutation, side chains of nearby negative residues are also shown. For the p.Leu401Pro mutation, nearby hydrophobic residues are shown.

We studied the molecular effects of these mutations in more detail using a homology model of the TGFB3 dimer. Asp263 is located in a surface loop where it is accessible for integrins (Figure 4). Mutation p.Glu216_Lys251del results in deletion of a central beta-strand and subsequent surface loop in the LAP domain (Figure 4). This will severely affect this domain’s conformation, including the position of the RGD motif, and thereby affect dimerization and inhibition of the TGFB3 domain.

Missense mutations p.Arg300Trp, p.Ile322Thr, and p.Leu401Pro are also predicted to alter TGFB3 function. Besides participating in the cleavage site, Arg300 is involved in several ionic interactions that will be lost with the substitution to tryptophan, and this bulky residue will likely induce steric rearrangements. Ile322Thr is predicted to alter the positioning of Arg325, an important amino acid residue for binding with TGFBR2. In addition, hydrophobic contacts with the N-terminal helix in the LAP domain will be affected by the substitution with threonine (hydrophilic). Substitution from Leu401 to proline is predicted to change the hydrophobic interactions with residues of both the LAP and TGFB3 domain.

The clinical phenotypes in the 10 additional families demonstrate significant overlap with Loeys-Dietz syndrome (Online Tables 3 to 6). Vascular involvement ranges from no cardiovascular abnormalities at age 64 (3-II:2) to type A (median age of 51 years, range 40 to 80 years) or type B aortic dissection (median age of 44.5 years, range 30 to 57), abdominal aortic dissection and death as a result of cerebral aneurysm dissection at age 55 (2-II:1) (Table 1). So far, no examples of early arterial dissection or dissection at small aortic dimension were observed. Other cardiovascular features include mitral valve disease, ranging from mild insufficiency to chorda rupture necessitating surgery, and persistent foramen ovale and atrial or ventricular septal defects. Disease beyond the aorta, with iliac and subclavian artery aneurysms, was only identified in 2 patients (1-II:12 and 1-III:13). No striking aortic or arterial tortuosity was observed.

**Table 1:** Patient Characteristics

	Affected Individuals (n = 43)∗
Sex, M/F	23/20
Age, yrs	34 (3–74)
Age at death, yrs	56 (40–80)
Age at dissection, yrs	47.5 (30–80)
Cardiovascular findings	
Type A dissections, age, yrs	4 (51; 40–80)
Type B dissections, age, yrs	6 (44.5; 30–57)
Aortic aneurysm†, age, yrs	6 (34; 3–68)
Abdominal aortic surgery‡	2
Disease beyond aorta§	3
Skeletal findings	
Tall stature	12
Arachnodactyly	16
Pectus deformity	8
Kyphoscoliosis	11
Joint hypermobility	9
Loeys-Dietz features	
Hypertelorism	14
Bifid uvula	11
Cleft palate	5

Values are n, median (range), or n (median; range).

∗ Not all patients were evaluated for all features.

† Four aneurysms affected the sinuses of Valsalva, and 2 only affected the ascending aorta.

‡ Surgery was performed on 1 patient at age 43 years and 1 at 50 years.

§ Cerebral, iliac, or subclavian arteries (n = 1 for each location).

Typical LDS findings such as hypertelorism, bifid uvula and cleft palate, cervical spine instability, and club foot deformity are commonly observed (Figure 2, Table 1, Table 2, and Online Tables 3 to 6). Other recurrent features include dolichocephaly, high-arched palate, retrognathia (with surgery in case 3-III:1), tall stature, joint hypermobility, arachnodactyly, pectus deformity, and inguinal hernia (Table 1, Figure 2). No evidence for ectopia lentis was found in the medical records. Early-onset osteoarthritis was only reported in 2 individuals (10-II:1 and 11-II:1). The clinical features from 43 identified patients belonging to 11 families are summarized in Table 1. We observed a striking intrafamilial and interfamilial clinical variability with typical LDS features in some, but complete absence in others.

**Table 2:** Comparison of Phenotypical Characteristics of Patients With *TGFBR1/2, SMAD3, TGFB2, and TGFB3* Mutations

Phenotype	*TGFBR1* *TGFBR2*	*SMAD3*	*TGFB2*	*TGFB3*
Hypertelorism	✓	✓	✓	✓
Bifid uvula/cleft palate	✓	✓	✓	✓
Exotropia	✓	✓	✓	✓
Craniosynostosis	✓	✓	×	×
Cervical spine instability	✓	✓	×	✓
Retrognathia surgery	✓	✓	✓	✓
Scoliosis/spondylolisthesis	✓	✓	✓	✓
Clubfoot	✓	✓	✓	✓
Osteoarthritis	✓	✓	×	✓
Dural ectasia	✓	✓	✓	?
Pneumothorax	✓	✓	✓	×
Hernia	✓	✓	✓	✓
Dissection at young age	✓	✓	✓	?
Disease beyond root	✓	✓	✓	✓
Cerebral hemorrhage	✓	✓	✓	✓
Arterial tortuosity	✓	✓	✓	×
Autoimmune findings	✓	✓	✓	✓

A check mark indicates presence of the clinical feature, an X indicates absence of the clinical feature, and a question mark indicates presence of a clinical feature is unknown.

**Figure 5:**
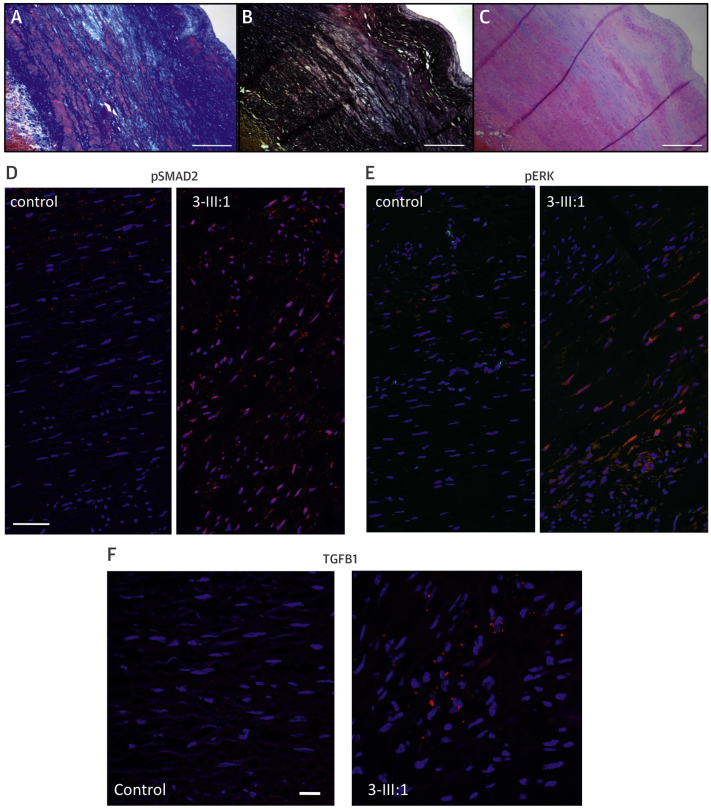
Cardiovascular Pathology and Immunohistochemical Analysis of TGFB Family Proteins in a Human Subject With *TGFB3* Mutation (3-III:1; p.Asp263His) **(A)** Masson trichrome staining shows increased deposition of collagen **(dark blue)** and loss of smooth muscle fibers **(red)** in the media. **(B)** Elastin stain (Elastica van Gieson) shows loss of elastin fibers **(black)**. **(C)** Hematoxylin-eosin staining shows deposition of proteoglycan **(light blue)** in the media. **(A–C)** Scale bar indicates 2 mm. **(D–F)** Cross sections of the media of the aortic wall of patient 3-III:1 and a matched control. Red staining corresponds to pSmad2 **(D)**; pERK **(E)**; and TGFB1 **(F)**. Scale bars indicate 50 μm **(D–E)**, 20 μm **(F)**. **Blue** staining shows cell nuclei (DAPI), colocalization is **purple**. **Red** staining not colocalized with DAPI is nonspecific.

We subsequently investigated the effect of the p.Asp263His mutation on aortic wall architecture and TGF-β signaling. Microscopic examination of the dissected aortic wall, obtained at the time of surgery (3-III:1), showed elastic fiber fragmentation with higher collagen and proteoglycan deposition (Figures 5A to 5C). These histopathological findings are highly reminiscent of both MFS and LDS (8). Retrieved pathology reports from 2 patients (1-II:4 and II:5) carrying the p.Glu216_Lys251del mutation (family 1) also described extensive elastic fiber fragmentation with “pseudo cyst formation” in the medial layer of the dissected aorta and “aortic medial degeneration.” In families 9 and 10, only mild elastic fiber fragmentation was observed.

To investigate TGF-β signaling in the aortic wall of a patient carrying a *TGFB3* mutation (p.Asp263His), we performed immunohistochemical analysis of aortic tissue (Figures 5D to 5F). Very similar to what has been detected in TGFB2-deficient aortic walls of humans and mice (8), we observed evidence of paradoxically enhanced TGF-β signaling in the aortic wall of a *TGFB3* mutant patient, as shown by increased pSMAD2 (canonical TGF-β signaling), pERK (noncanonical TGF-β signaling), and elevated *TGFB1* messenger RNA (Figures 5D to 5F).

## Discussion

During mouse embryonic development, *Tgfb3* is expressed in several tissues, including cardiovascular, pulmonary, skin, and craniofacial structures. Although *Tgfb3* is expressed in overlapping fashion with *Tgfb2* in the cardiovascular system, most attention has been paid to its role in palatogenesis, as *Tgfb3* knockout mice die at birth because of cleft palate 38, 39. No major cardiac developmental defects have been reported in *Tgfb3*-deficient mice 38, 40, 41. Although minor abnormalities at the aortic arch level, as well as in position and curvature of the aortic arches and myocardial architecture, were described in the *Tgfb3* knockout mice, no data are available on the aortic sizes of conditional knockout or haploinsufficient animals 39, 40. Of interest, the presence of aortic aneurysms and ruptures recapitulating the human phenotype were previously overlooked upon the initial phenotypic description of *Tgfb2* haploinsufficient and *Smad3* knockout mouse models 8, 30.

Because we observed 3 truncating mutations and an in-frame splice site mutation, we hypothesize that the *TGFB3* mutations lead to loss of function (LOF) of TGFB3. In addition, 2 missense mutations, located in the LAP domain, alter critical residues that are relevant for TGFB3 activation by integrins and TGFB3 processing 35, 42. Mice carrying a missense mutation affecting the RGD integrin-binding motif of Tgfb1 recapitulate the phenotype of Tgfb1 knockout mice (43), suggesting that the *TGFB3* mutation disrupting the RGD (p.Arg263His) might also lead to LOF. Similarly, molecular analyses and predictions based on the TGFB3 dimer model confirm that most *TGFB3* mutations reported here cause LOF. Although it was previously hypothesized that patients with LOF mutations in *TGFB3* lack cardiovascular phenotypes (16), we clearly demonstrate that *TGFB3* LOF mutations associate with aortic and other arterial aneurysms/dissections and mitral valve disease, and recognize an extremely variable cardiovascular phenotype in the *TGFB3* cohort described here. The relatively young age of previously reported patients with *TGFB3* mutations (8 [16] and 10.5 years of age [17]) might explain the lack of obvious cardiovascular disease. On the basis of expression studies of the mutant TGFB3 protein in a Xenopus model, Rienhoff et al. (16) hypothesized that the mutated, inactivated allele (p.Cys409Tyr) leads to a nonfunctional protein, decreasing both canonical and noncanonical TGF-β signaling. By contrast, our experiments on human aortic tissue reveal a signature of increased TGF-β signaling. These findings confirm our prior experience that mutational hits in the TGFBR1/2 receptors, the SMAD3 signal transducer, or the TGFB2 ligand lead to a paradoxical increase in TGF-β signaling, as evidenced here by increased immunohistochemical signals for pSMAD2, pERK, and TGFB1 5, 7, 8. Shifts in balances between canonical (pSMAD2) and noncanonical (pERK) cascades, classic, and alternative (BMP-driven) TGF-β superfamily cascades, as well as shifts in ligand expression (TGFB1 vs. TGFB2 or TGFB3) seem likely to be important contributing factors 44, 45.

*TGFB3* mutations also appear to have opposing effects on height, as 1 patient in this study (3-III:1, p.Asp263His) has short stature and received growth hormone therapy during puberty, and the patient reported by Rienhoff et al. (16) (p.Cys409Tyr) presented with short stature (5th percentile), whereas others (several patients in this study and the patient reported by Matyas et al. [17]) presented with tall stature. *TGFB3* mutations affecting residue Arg300 are associated with cleft palate and/or bifid uvula in our patients (Online Tables 3, 4, and 6) and in the patient reported by Matyas et al. (17). Our study confirms the association of *TGFB3* mutation with overt cleft palate in humans and endorses its important role in palatogenesis.

Although our experience is limited to 43 patients in 11 families, our findings warrant comprehensive cardiovascular imaging of the patients. Thus far, no strong evidence has emerged for early aortic dissection in *TGFB3* mutant patients, but as the phenotypical spectrum associated with *TGFBR1/2, SMAD3,* and *TGFB2* has now been demonstrated to be extremely wide, we cannot rule out the occurrence of early catastrophic events. We recommend yearly echocardiographic evaluation of the aortic root in all mutation carriers, complemented with at least 1 baseline imaging of the complete aorta and side branches. Frequency of follow-up should be guided by initial findings, family history, and experience still to be gained. Depending on family history and future knowledge, additional imaging of the brain vessels might be indicated. Furthermore, the true incidence and full spectrum of autoimmune manifestations in *TGFB3* mutation carriers should be determined in follow-up studies.

### Study limitations

Not all clinical features are acquired in all patients. Further studies are needed to fully characterize the phenotypical spectrum we identified here. The predicted effects of the mutations in the homology model of TGFB3 are theoretical and should be complemented with additional protein studies, and the immunohistochemistry studies are hampered by limited availability of patients' aortic wall tissues.

## Conclusions

We demonstrate that mutations in the *TGFB3* ligand are responsible for a syndromic form of aortic aneurysmal disease. Consistent with our previous findings in *TGFBR1/2*, *SMAD3,* and *TGFB2* mutation carriers, our study also provides evidence for a paradoxical increase in TGF-β signaling in the aorta. The clinical histories of the patients in our cohort warrant lifelong and widespread cardiovascular surveillance in patients with *TGFB3* mutations (Central Illustration). Further research explaining the wide clinical variability is strongly indicated.

**Central Illustration:**
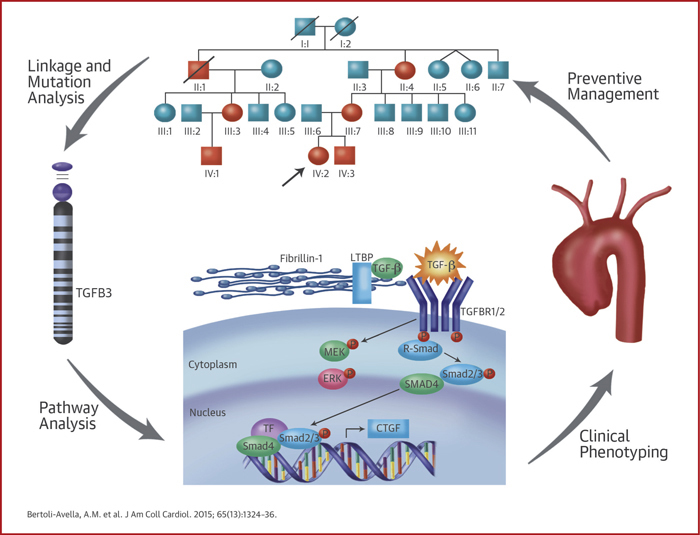
The Pathway From Patient to Gene and Back The figure summarizes how initial identification of patients and families, followed by linkage and mutation analysis led to the discovery of *TGFB3* mutations. Further exploration of the TGF-β pathway allowed a better phenotypical delineation and characterization that will have implications in personalized clinical management.

Perspectives **COMPETENCY IN MEDICAL KNOWLEDGE:** Mutations in genes encoding components of the TGF-β signaling pathway can cause aortic and arterial aneurysm and dissection.

**COMPETENCY IN PATIENT CARE 1:** Screening for *TGFB3* mutations should be added to the expanding array of genetic testing for patients with unexplained aortic aneurysmal disease or arterial dissection.

**COMPETENCY IN PATIENT CARE 2:** Vascular imaging should be extended beyond the aortic root in patients with genetic mutations affecting TGF-β signaling because aneurysmal disease may involve more distal portions of the aorta and its arterial branches.

**TRANSLATIONAL OUTLOOK:** Further studies are needed to assess the safety and efficacy of such treatments as angiotensin receptor blocking drugs, which inhibit TGF-β activity, for prevention of aortic and arterial aneurysm expansion and dissection in patients with mutations involving TGF-β signaling pathways.
